# The ultimate database to (re)set the evolutionary history of primate genital bones

**DOI:** 10.1038/s41598-021-90787-2

**Published:** 2021-05-27

**Authors:** Federica Spani, Maria Pia Morigi, Matteo Bettuzzi, Massimiliano Scalici, Gabriele Gentile, Monica Carosi

**Affiliations:** 1 Diagnostic Imaging Unit, Departmental Faculty of Medicine and Surgery, Campus Bio-Medico University of Rome, Rome, Italy; 2 Department of Sciences, Roma Tre University, Rome, Italy; 3 Department of Physics and Astronomy, University of Bologna, Bologna, Italy; 4 National Institute of Nuclear Physics, Rome, Italy; 5 Department of Biology, University of Rome Tor Vergata, Rome, Italy

**Keywords:** Evolutionary theory, Phylogenetics, Data acquisition, Data integration, Databases

## Abstract

Scientific literature concerning genital bones in primates consists of both ancient works (dating back to the nineteenth century) and more recent revisions/meta-analyses, which, however, are not always so detailed or exhaustive. Based on a thorough analysis, several conflicting data, inaccurate references, and questionable claims have emerged. We generated a binary matrix of genital bone occurrence data, considering only data at the species level, based on (1) a rigorous literature search protocol, (2) raw data (collected exclusively from primary literature), (3) an updated taxonomy (often tracing back to the species taxonomic history) and (4) new occurrence data from scanned genitals of fresh and museum specimens (using micro-computed tomography-micro-CT). Thanks to this methodological approach, we almost doubled available occurrence data so far, avoiding any arbitrary extension of generic data to conspecific species. This practice, in fact, has been recently responsible for an overestimation of the occurrence data, definitively flattening the interspecific variability. We performed the ancestral state reconstruction analysis of genital bone occurrence and results were mapped onto the most updated phylogeny of primates. As for *baculum*, we definitively demonstrated its simplesiomorphy for the entire order. As for *baubellum*, we interpreted all scattered absences as losses, actually proposing (for the first time) a simplesiomorphic state for the clitoral bone as well. The occurrence data obtained, while indirectly confirming the *baculum/baubellum* homology (i.e., for each *baubellum* a *baculum* was invariably present), could also directly demonstrate an intra-specific variability affecting *ossa genitalia* occurrence. With our results, we established a radically improved and updated database about the occurrence of genital bones in primates, available for further comparative analyses.

## Introduction

The metaphor ‘standing on giants’ shoulders’ perfectly elucidates the main aim of a literature review^1^. The metaphor was transferred to scientific knowledge by Isaac Newton^2^ who, in a letter to Robert Hooke in 1675, wrote: “If I have seen further it is by standing on the shoulders of Giants”^3^. Therefore, when reconstructing the accumulated knowledge (i.e., the ‘giant’) in a specific domain, the search of the original literature represents the first fundamental and crucial step of a study which likely allows to “see beyond”^4^.

One of the first mentions of both *baculum* and *baubellum* in primates was in 1871 by the French naturalist Alfred Grandidier^5^. Afterwards, several authors dedicated their studies to the genital anatomy in primates^6^ and some of them were specifically interested in genital bones^7–25^. Recently, some authors shifted the attention from the descriptive anatomy to the evolution and adaptive meaning of such bones in several mammal orders, primates included^26–29^. Based on different datasets and analyses, these studies obtained conflicting results.

The wide distribution of *baculum* occurrence throughout the order of Primates had been interpreted as a primitive condition^18–20,25,30^, nevertheless, the first phylogenetic reconstructions of the character state for genital bones have not been available until Schultz et al.^26^, and in the same year, immediately afterwards, Brindle and Opie^28^. Within a comparative framework, the rationale of the study of Schultz et al.^26^ was to investigate the inconsistency of hypotheses based on functional explanations and selective forces driving the evolution of *baculum* in mammalian class^20,31–44^. In their study, Schultz et al.^26^ found an almost equal number of gains and losses of the *baculum* (9 gains, and 10 losses), to finally conclude that *bacula* are indeed not homologous structures in mammals. This conclusion, the authors stated, might well support the difficulty in finding a shared function and evolution of *bacula* among *taxa*. Though not focused on the primate order, the study by Schultz et al.^26^ presented an unresolved ancestry for *baculum* in this clade. However, the presence of *baculum* as an ancestral condition in primates was found by Brindle and Opie^28^, who went even further, suggesting that *baculum* first arose between the split of non-placental/placental mammals and the most recent common ancestor of primates and carnivores.

The female counterpart of male penile bone, the *baubellum,* although targeted by several publications on several mammalian *taxa*^10,45–51^ could still be labeled as a neglected topic in recent primatology^52^. In fact, no hypotheses about primate *baubellum* evolution have been put forward, and only accounts of its occurrence were reported^5,7,8,10,11,16,17,21,23,45,53–63^ together with mentions of its developmental homology with the *baculum*^18,19,25,57,64,65^ (the latter, however, experimentally demonstrated for non-primate mammals only^66,67^). The limited data about *baubellum* in primates were almost all confined to the anatomical records in old publications (e.g.﻿^7,8,10,11,16^). The most recent new data goes back to 2001^23^.

Only recently, occurrence data for *baubellum* in mammals have been used to investigate the evolutionary history of the trait in a phylogenetic framework^29^. Findings, though referred to mammals in general, were useful twofold for the present study: (i) in the same species, *baubellum* occurrence matched 100% with *baculum* occurrence and in no species, *baubellum* presence matched with *baculum* absence); (ii) no evolutionary pattern, in terms of ancestry, was found, except what has been interpreted as a *baubellum* evolutionary “lability”, based on the significantly higher number of gains and losses if compared to the *baculum* data. Thus, although primates were not the specific focus of the discussion, the few data analyzed would suggest both a *baculum* and a *baubellum* ancestry in primates (23 out of 27 primate species with occurrence data for both genital bones^29^).

Conflicting results among studies might be partly a consequence of the use of different analytical approaches. Nevertheless, our initial inspection of genital bone presence/absence datasets used^26–29^ drew our attention to unexpected differences found in both sample size and references used. We hypothesized three possible factors contributing to this: (a) different data retrieval strategies were probably adopted, therefore affecting sample size and generating some of the inconsistencies found between datasets (e.g., conflictual occurrence data and inaccurate data-reference match), (b) sample size was often (but not always) constrained by the aims of the study (e.g., *baculum* in association to *baubellum* data^29^; *baculum* associated to penile spine data^27^), and (c) the different phylogenetic frameworks in which the analyses were performed (a mammal supertree, including the primate phylogeny by Perelman et al.^68^, used by Schultz et al.^26^ and Lough-Stevens et al.^29^; a posterior distribution of 10,000 molecular Bayesian MCMC phylogenies^69^ for analyses on primates used by Brindle and Opie^28^).

When the target topic is anatomy, primary literature mostly consists of old publications, scientific treatises, and books. The information therein can be dispersed and retrieving data may be challenging. For example, Burt^48^ dated the discovery of genital bones in primates approximately in the Seventeenth Century, and no reference was given. Efforts in considering primary literature may prevent pitfalls that might jeopardize an exhaustive dataset construction such as (i) the involuntary omissions, miscitations or misinterpretations of more recent and easily accessible reviews, and (ii) all controversial cases of taxonomical incongruences and conflicting data at the species level due to taxonomic reviews of the primate order across the years. With this work, we took one step backward from current conclusions of studies of genital bone evolution in primates and firstly redefined the bone occurrence dataset by relying on original primary literature data only. By doing so we aimed at compensating involuntary inaccuracies. Secondly, we acquired new original micro-CT data (coming from both fresh and museum specimens), and the enforced database resulting from the combination of the two data sources was finally used to reconstruct the ancestral character state of both *baculum* and *baubellum* in primates.

## Results

After a thorough literature search, we obtained data of genital bone occurrence, at either genus or species level shown in Supplementary Table S1. The scanning of original samples by museum and fresh specimens supported the occurrence of genital bones in 29 primate *taxa* never investigated so far (Supplementary Table S2). Summing up data deriving from both literaure and scanned samples we finally obtained an occurrence database including N = 280 species for *baculum*, and N = 78 species for *baubellum* (see below).

### *Baculum* and *baubellum* occurrence databases

Strictly at the species level, literature search allowed to record 25 absences, 242 presences, and one doubtful case for the *baculum* (N = 268 species), and 13 absences, 42 presences, and 3 doubtful cases for the *baubellum* (N = 58 species). Likewise, micro-CT scanning allowed to record 6 absences, 9 presences, and 4 doubtful cases for the *baculum* (N = 11 species and one subspecies; N = 12 additional species with new data), and 25 absences and 1 presence for the *baubellum* (N = 19 species and one subspecies; N = 20 additional species with new data).

Our *baculum* occurrence dataset (literature plus scanned sample data) covered 60% of extant primate species (N = 280 out of N = 467) recognized by the International Union for Conservation of Nature Red List Data (IUCN Red List^70^). The remaining primate species (i.e., 40%) were classified as ‘omitted data’. Our *baubellum* occurrence dataset (literature plus scanned sample data) covered 16.7% of extant species (N = 78 out of N = 467) recognized by IUCN Red List^70^. The remaining primate species (i.e., 83.3%) were classified as ‘omitted data’. For both genital bones, species classified as “omitted data” included: (a) those present in the literature, only described at the genus level; (b) those present in the literature (e.g., in anatomical treatises, either as dissected specimens or as figures) for which, however, neither presence nor absence of the *baculum* was reported (i.e., *omissis*); and (c) those absent both in the literature and in museum collections explored for the present study.

**Figure 1:**
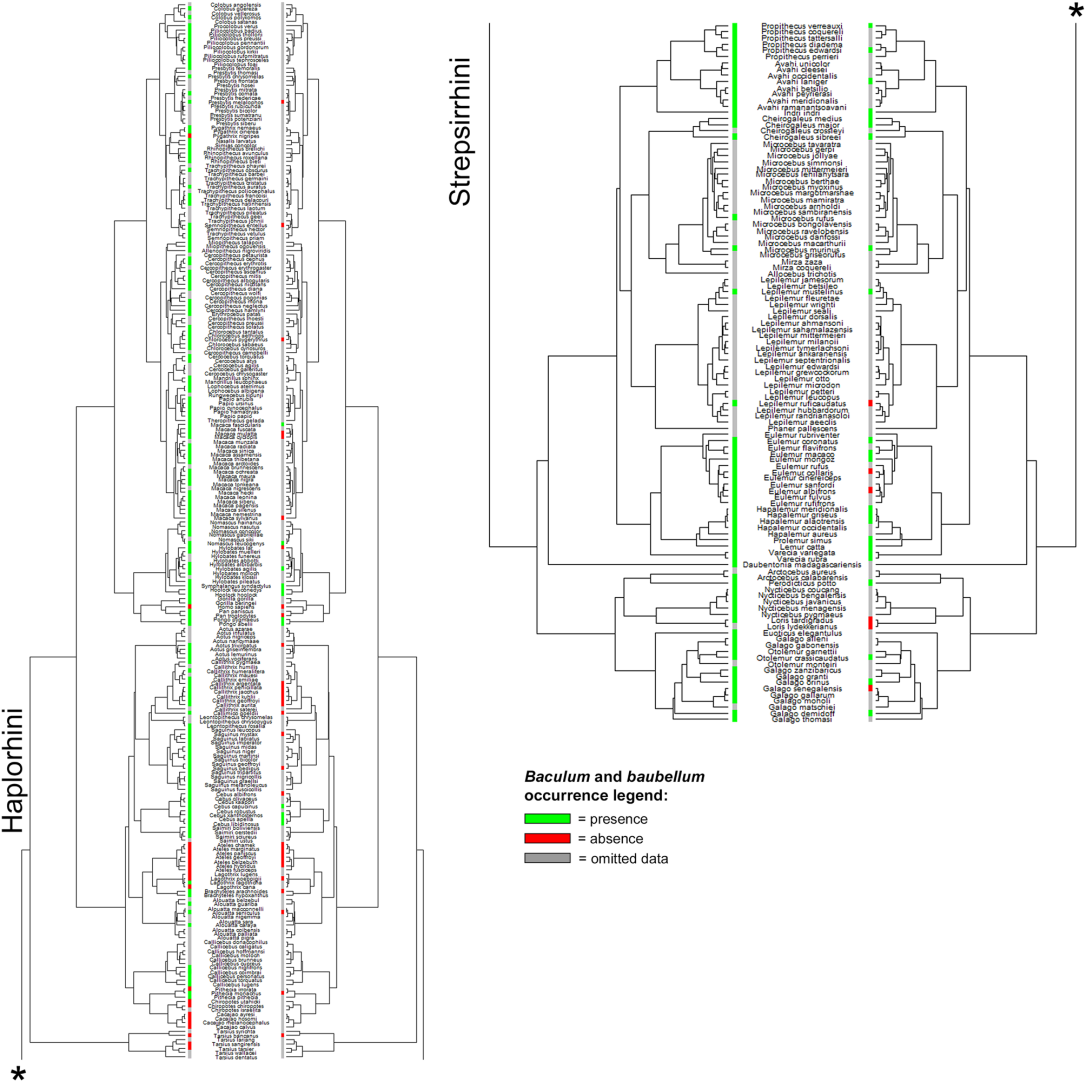
State of the knowledge of *baculum* and *baubellum* occurrence. Comparison between our *baculum* (left) and *baubellum* (right) occurrence data coverage of primate phylogeny. Green lines indicate the presence of *ossa genitalia*. Red lines indicate the absence of *ossa genitalia*. Grey lines stand for ‘omitted data’ (i.e., all those species for which neither absence nor presence of genital bones was ever stated). Phylogeny by Springer^71^, dropped. Figure generated in R (v. 4.0.3, https://www.R-project.org/)^114^.

Figure 1 shows the distribution of genital bone occurrence data mapped onto Springer’s phylogeny^71^, therefore providing a quick glimpse at our data coverage throughout the primate order, comparing *baculum* and *baubellum* data.

### Ancestral character state analysis: *baculum* and *baubellum* stochastic character mapping

The D statistics for both *baculum* and *baubellum* showed a strong phylogenetic signal (Table 1).

**Table 1:** D statistic for phylogenetic signal.

Phylogeny	N states		N permutations	D	p_1_	p_0_
	AB	PR				
*Baculum*						
Springer	22	222	1000	− 0.18	0	0.77
TimeTree	19	205	1000	− 0.28	0	0.82
*Baubellum*						
Springer	37	35	1000	0.09	0	0.38
TimeTree	33	33	1000	0.07	0	0.42

Calculation of D statistic for the phylogenetic structure of two binary variables (i.e., *baculum* and *baubellum*) considering both phylogenies by Springer^71^ and Timetree^72^. Counts of states (either absence—AB or presence—PR), total number of permutations (N permutation), sum of changes in estimated nodal values of both binary traits along edges in the phylogeny (D), a p value giving the result of testing whether D is significantly different from one (p_1_), a p value giving the result of testing whether D is significantly different from zero (p_0_).

**Figure 2:**
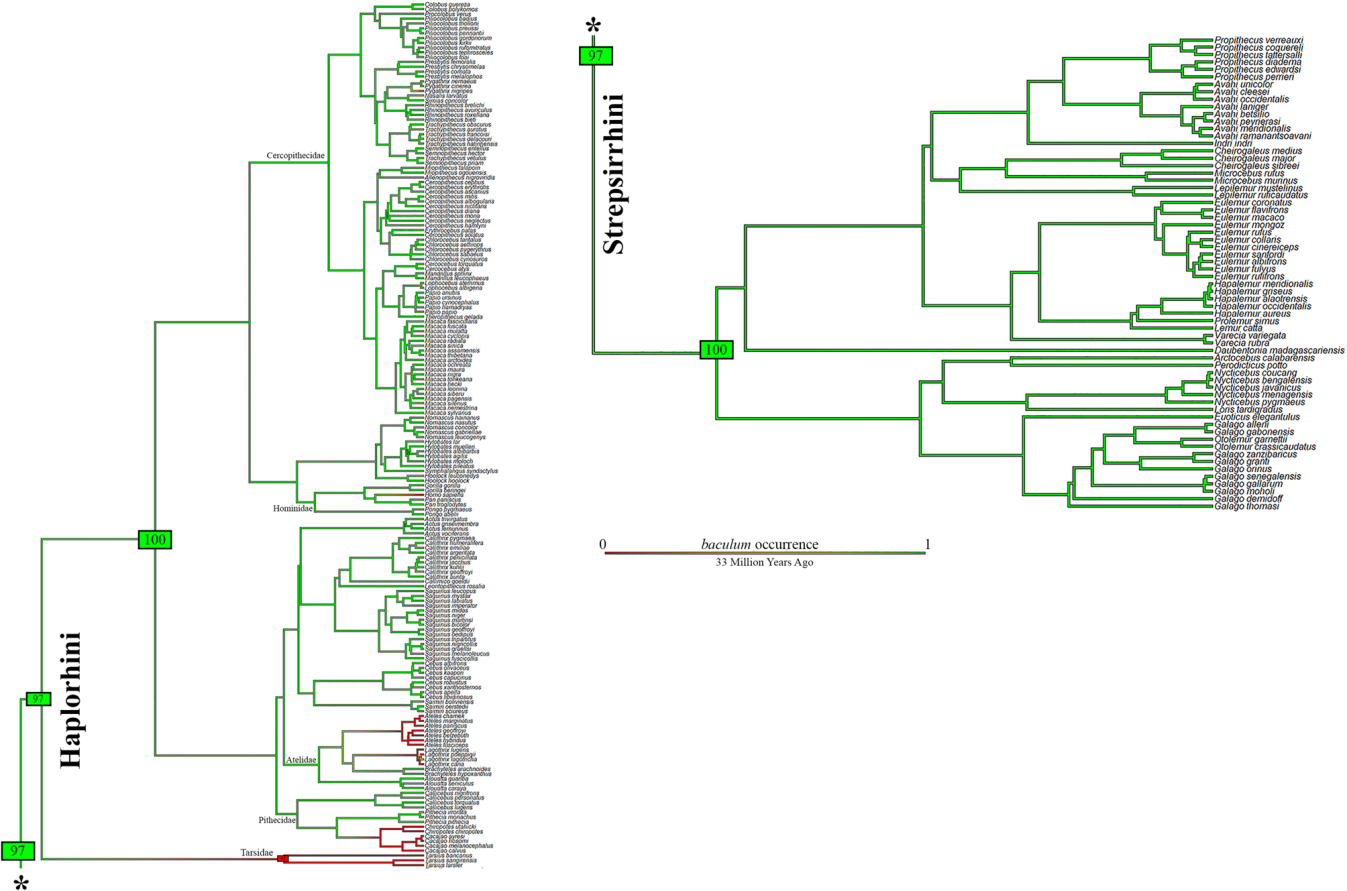
Ancestral character state reconstruction of *baculum* excluding outgroups. Results from 1000 stochastic character maps (where the character analyzed is the *baculum* occurrence) displayed in aggregate (outgroups were excluded from the analysis). The colour of edges in the tree gives the posterior probability (computed as the relative frequency across stochastic maps) of each *baculum* state through the history of the clade. Green indicates a high posterior probability of *baculum* presence and numbers in green (or red) boxes indicate the proportion of iterations that mapped *baculum* occurrence to those particular branches. The length of the legend also gives a scale for the tree branch length (in this case in Millions of Years Ago). Phylogeny by Springer^71^, dropped. Figure generated in R (v. 4.0.3, https://www.R-project.org/)^114^.

Results of the first stochastic mapping analysis for *baculum* (including five outgroups, see Supplementary Fig. S3 for a coloured tree) indicated a mean state change equal to 11. Changes were of two kinds: (1) from absence to presence (2.4 times); (2) from presence to absence (8.8 times). The mean total time spent in each state was 13.4% for absence and 86.6% for presence. Based on this, *baculum* was gained at least 2 times and was lost at least 8 times in at least 50% of the 1000 iterations of stochastic mapping. Results from the second analysis (not including five outgroups) showed a mean state change of 8.8 between *baculum* presence and absence. The Springer’s and Timetree phylogenies^72^ produced almost identical results (Supplementary Table S4, Supplementary Fig. S5). The mean total time spent in each state was 8.2% for absence and 91.8% for presence. Thus, *baculum* evolved just once (or twice) along primate phylogeny (character state change from absence to presence) and was lost 7 times (character state change from presence to absence) in at least 50% of the 1000 iterations of stochastic mapping. The root of the coloured tree (Fig. 2) appeared as total-green, attesting to a high probability of *baculum* presence in the primate ancestor. Losses involved 5 families: Tarsidae (the few species available); Pithecidae (*Pithecia irrorata* Gray, 1842, and the *Chiropotes* + *Cacajao* clade); Atelidae (*Ateles* clade and *Lagothrix* clade—if we exclude the doubtful case of *L. lagothricha* [Humboldt, 1812]); Hominidae (*Homo sapiens* Linnaeus, 1758); Cercopithecidae (*Pygathrix nigripes* [A. Milne-Edwards, 1871]).

**Figure 3:**
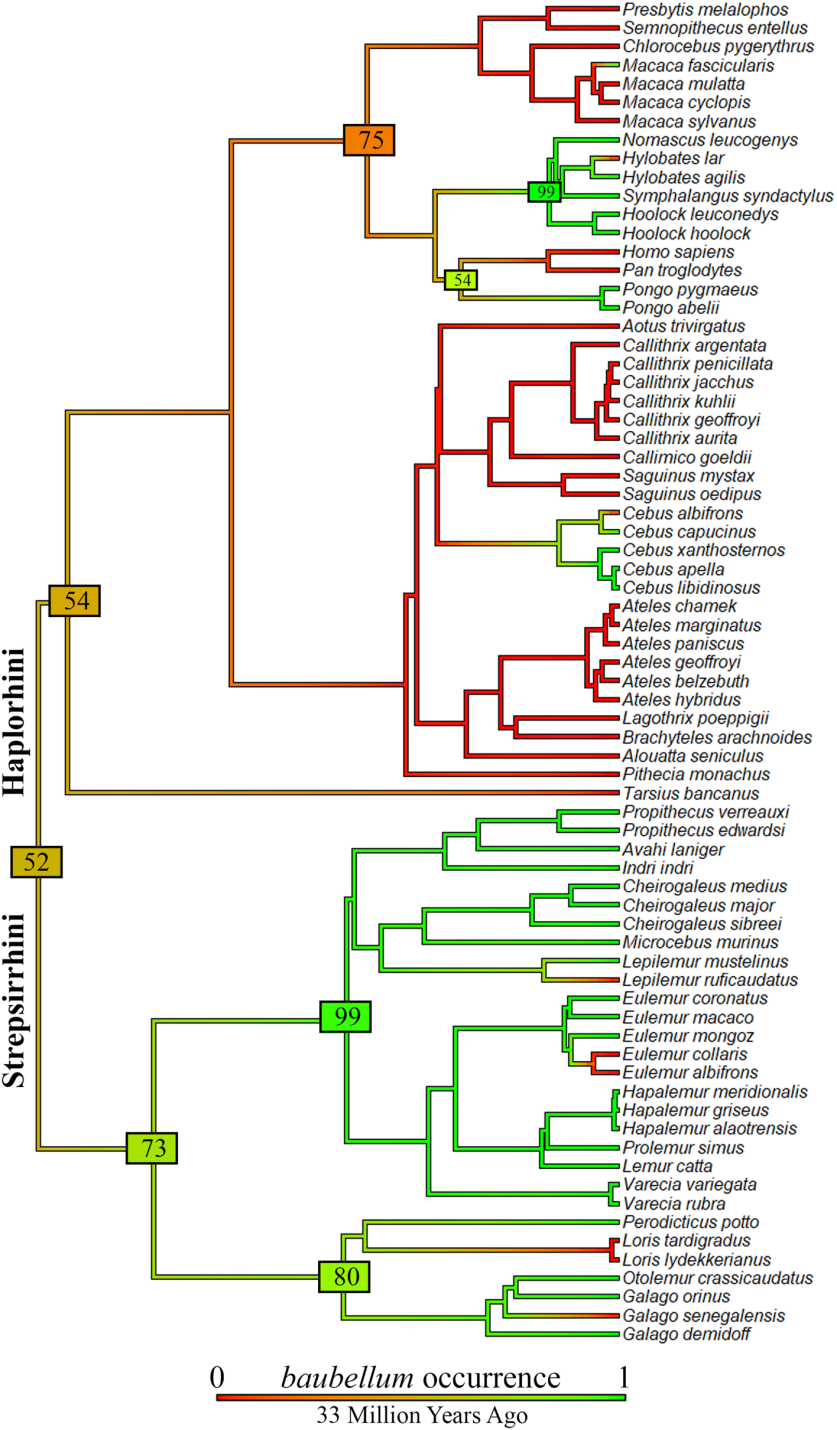
Ancestral character state reconstruction of *baubellum* excluding outgroups. Results from 1000 stochastic character maps (where the character analyzed is the *baubellum* occurrence) displayed in aggregate (outgroups were excluded). The colour of edges in the tree gives the posterior probability (computed as the relative frequency across stochastic maps) of each *baubellum* state through the history of the clade. Green indicates a high posterior probability of *baubellum* presence and numbers in green (or light green) boxes indicate the proportion of iterations that mapped *baubellum* presence to those particular branches. The length of the legend also gives a scale for the branch tree length, (in this case in Millions of Years Ago). Phylogeny by Springer^71^, dropped. Figure generated in R (v. 4.0.3, https://www.R-project.org/)^114^.

Similarly, results of the first stochastic mapping for *baubellum* (including five outgroups, see Supplementary Fig. S6 for a coloured tree) indicated a mean state change equal to 19.3. From absence to presence, character changes occurred at least 8 times, whereas from presence to absence, changes occurred at least 11 times. The mean total time spent in each state was 52.97% for absence and 47.03% for presence. Based on this, *baubellum* was gained 8 times and was lost 11 times in at least 50% of the 1000 iterations of stochastic mapping (see Supplementary Fig. S6), sufficient to reach convergence. Results from the second analysis showed a mean state change of 16.7. The mean total time spent in each state was 46.34% for absence and 53.6% for presence. According to these data, *baubellum* evolved 7 times along primate phylogeny and was lost 9 times in at least 50% of the 1000 iterations of stochastic mapping. Figure 3 reports the coloured tree. While haplorrhine species showed a shared absence of *baubellum*, except for *Cebus* genus and Hylobatidae family, strepsirrhine species showed a clear ancestry of *baubellum,* except for three punctual losses observed (*Lepilemur ruficaudatus* A. Grandidier, 1867, *Loris* clade and *G. senegalensis*). With respect to the Springer’s tree^71^, the Timetree phylogeny^72^ produced slightly higher mean of state changes, but almost identical results when transitions were plotted on the tree (Supplementary Table S4, Supplementary Fig. S7).

**Figure 4:**
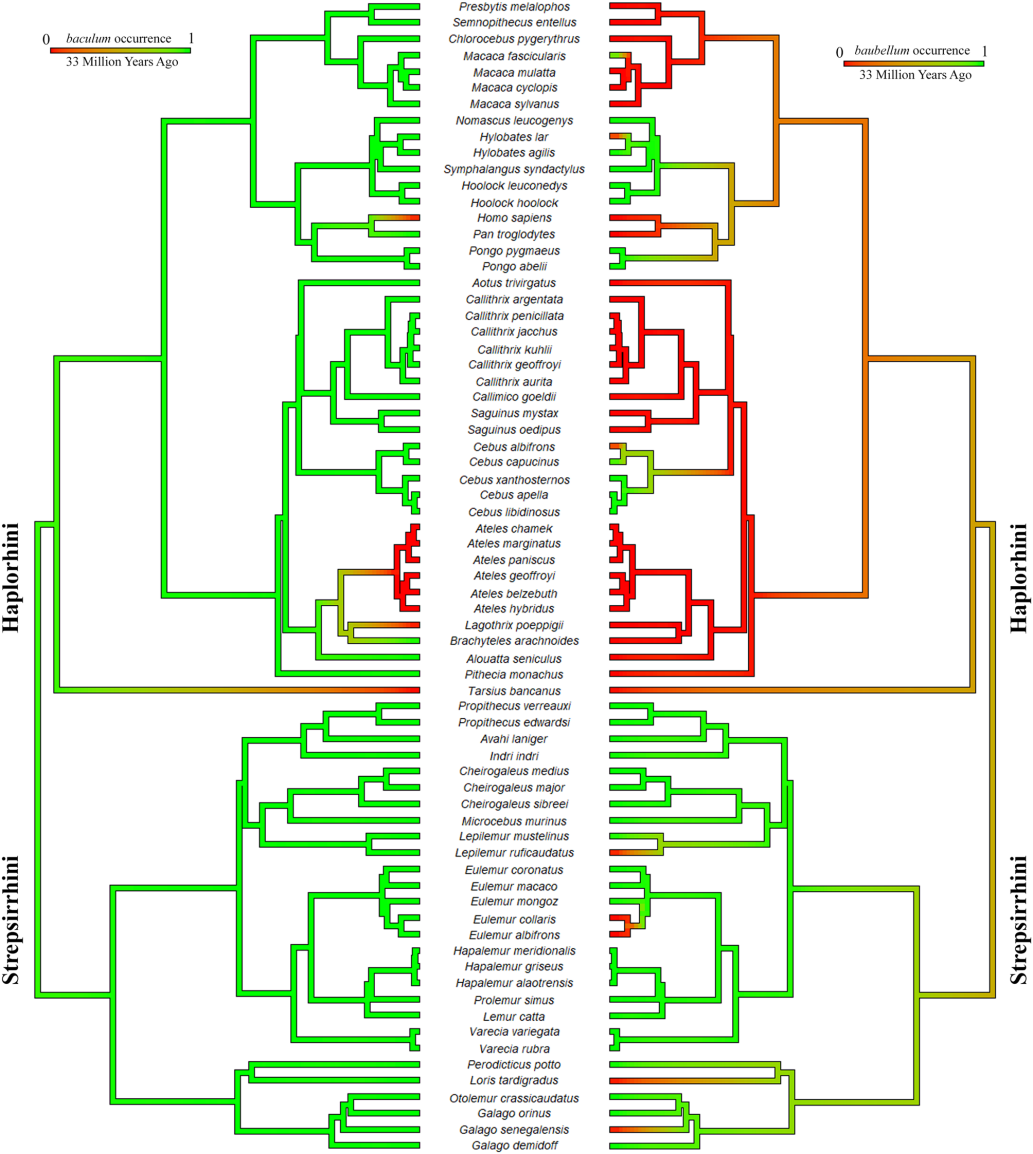
Comparison between *baculum* and *baubellum* ancestral character state reconstructions, displayed in aggregate. In this case, only primate species having both *baculum* and *baubellum* data were selected to make a comparison. Green indicates a high posterior probability of *ossa genitalia* presence. The length of the legend also gives a scale for the branch tree length (in this case in Millions of Years Ago). Phylogeny by Springer^71^, dropped. Figure generated in R (v. 4.0.3, https://www.R-project.org/)^114^.

Finally, by comparing data on *baculum* and *baubellum,* we found an occurrence overlap corresponding to 71 primate species out of 72 species for which *baubellum* occurrence data were available. In this case, *Loris lydekkerianus* Cabrera, 1908 was excluded because it was the only species for which the *baculum* occurrence was omitted (Fig. 4). Our dataset confirmed that for all species having a *baubellum*, a *baculum* was always recorded while the opposite was not always the case. Our dataset confirmed also that a species having a *baubellum* but no *baculum* is yet to be found.

## Discussion

Our data search strategy, the use of primary literature, cautious criteria for attributing occurrence data to the species, and the high number of micro-CT scanned specimens, allowed the generation of a large and robust dataset for both *baculum* and *baubellum* occurrence in primates, with data rigorously verified at the species level. Such a dataset added new species to those used by the most recent and extensive studies on *baculum* and *baubellum* evolution published so far (for *baculum*, additional 176 species to Schultz et al.^26^; additional 178 species to Brindle and Opie^28^; for *baubellum*, additional 51 species to Lough-Stevens et al.^29^) and allowed us to finally demonstrate the symplesiomorphy of the *baculum* in the entire order of Primates^18–20,25,28,30^. With a more complex (and unresolved) picture, we also may hypothesize the ancestry of the *baubellum* limited to the strepsirrhines.

Our results, irrespectively of phylogeny used and although relying on a different dataset, well support *baculum* ancestry in the primate order stated in Brindle and Opie^28^, however, our data contrast the uncertain ancestral state found by Schultz et al.^26^. In the latter, two of 9 independent trait transitions of *baculum* in mammals, are the gains found in primates, namely in Strepsirrhini and Simiiformes, which prevented from resolving the ancestry. We also found at least two possible gains of *baculum* in our first analysis of *baculum* transitions (outgroups included). The second analysis (outgroups excluded), however, showed that only one of those two *baculum* acquisitions affected the common primate ancestor, while the other occurred outside the primate order (in fact, the total number of acquisitions decreased by excluding outgroups). In conclusion, what emerged from our results was a single evolution at the base of the primate tree and 8 losses afterwards.

Among catarrhines, *H. sapiens* and *P. nigripes* seemed to be the only species without a *baculum*. The presence of a bone in the human penis has been reported several times^73–77^, however, whereas interpreted in the earlier literature as a constant morphological character appearing intermittently^78^, the bone was subsequently invariably considered (and described) as a pathological condition^73–76^. Since no fossils of human penile bone have been found until now, the loss of *baculum* could be considered a distinctive trait for the entire genus. Not so for *Pygathrix* genus, in which the *baculum* presence has been reported for two species: in *P. nemaeus* (Linnaeus, 1771)^20^ and *P. cinerea* Nadler, 1997, as an inferred datum (it was considered a subspecies of *P. nemaeus* in the past; present study).

In platyrrhines, those families (i.e., Atelidae and Pitheciidae) known to include *taxa* with an overall tendency to *baculum* size reduction (compared to Catarrhines^18–20,26,59^), also showed most of the absences reported in the primate order. In the two Atelidae subfamilies, losses were limited to Atelinae, with a long-known absence of *baculum* in the *Ateles* clade, and a presence/absence condition in the *Brachyteles* + *Lagothrix* clade. The old and only record about *baculum* presence in *Brachyteles*^79^, has been recently confirmed by dissection of a hybrid *B. hypoxanthos* × *B. arachnoides*^24^, while the case of *Lagothrix* was somewhat more controversial. Pocock^12^ verified the absence of *baculum* in *Lagothrix poeppigii* Schinz, 1844 (= *L. infumata*) and, both Hill^57^ and Dixson^80^ confirmed the absence for other three congeneric species, that were *L. lugens* Elliot, 1907, *L. flavicauda* (Humboldt, 1812) (= *Oreonax flavicauda*), and *L. lagothricha*. For the latter, however, Machida and Giacometti^62^ made a general statement, reporting “All primates thus far studied [*including a sample of L. lagothricha, ndr*] have a short rod of bone or cartilage, the *baculum*, at the distal end of the septum that joins the two corpora cavernosa penis” (p. 50). Neither explanations nor species distinctions between either bone or cartilage were present anywhere in the paper. Although we included this presence in our trait transition analysis, not to lose the datum, it is nevertheless strongly recommended the exploration of additional *L. lagothricha* specimens to confirm that general statement. In the two Pithecidae subfamilies, losses were limited to Pithecinae, with *baculum* lacking in the monophyletic clade of *Chiropotes* and *Cacajao,* and one punctual loss reported in only one out of five *Pithecia* species *(P. irrorata*^19^; see Supplementary Table S1).

Finally, Tarsidae showed an absence of *baculum* (100% of posterior probability that their ancestor didn’t have a *baculum*) despite its position in the primate phylogeny (i.e., as the sister group of Simiiformes showing a 100% of posterior probability that the common ancestor had a *baculum*). Their condition of *baculum* absence was well supported in the literature by different authors that clearly stated the absence of bone inside the penis, supporting their data with dissected specimens^11,81,82^.

If limited to *baculum* losses, our results well overlapped with those reported in Schultz et al.^26^ (*H. sapiens*, *Ateles* clade, *Lagothrix* clade, *Cacajao* + *Chiropotes* clade, and *Tarsius* clade). In addition, for the 99 species shared by both Schultz’s and our datasets (representing the whole data analyzed by Schultz and collaborators^26^), we only differed in: (a) attributing *baculum* presence in *L. lagothricia* (Machida and Giacometti^62^ reported a presence—see above—while Schultz and collaborators^26^ reported an absence based on Hill^83^; (b) a few discordances in the taxonomy, that we based on IUCN Red List^70^ and ITIS (Integrated Taxonomic Information System^84^) (e.g., since 2005, *Cacajao rubicundus* is no longer considered a species but a subspecies of *Cacajao calvus* (I. Geoffroy Saint-Hilaire, 1847); since 2002, *Cebus apella* turned into *Sapajus* genus; since 2005 *Cercopithecus aethiops* turned into *Chlorocebus* genus; since 2001, *Galago demidovii* turned into *Galagoides demidoff* (G. Fischer, 1806); correct spelling for *Mico argentata* is *Mico argentatus* (Linnaeus, 1771); since 2005, *Presbytis senex* turned into a subspecies of *Semnopithecus vetulus* (Erxleben, 1777); and (c); considering that the datum reported for *T. spectrum*^81^ actually belongs to *Tarsius tarsier* (Erxleben, 1777; *T. spectrum* is no longer used), while Schultz et al.^26^ attributed it to *Tarsius syrichta* (Linnaeus, 1758) instead (the only *Tarsius* species present in the phylogeny they used).

More complex is the comparison of our results with those of Brindle and Opie^28^ whose dataset of 301 species was based on 4 references (8.7% out of 46 references of ours). A detailed comparison with our dataset and associated literature showed that their data unlikely reflect the state of knowledge about the occurrence of primate *bacula*. In fact, only 32% of their data (N = 97 species) are reported in their cited literature (and overlapped with our occurrence data), while the remaining 68% (N = 204 species) are actually not reported in their cited literature at the specific level. Moreover, this last portion of data mostly corresponds to species that are actually data deficient (*omissis*) in the literature. By using the literature data inclusively (e.g., family or genus data extended to all species of that taxon) Brindle and Opie^28^ involuntarily brought down the omitted data in the literature from N = 117 species (as resulting from our search) to only N = 49 species. We do not recommend this practice. By doing so, the risks of data inflation (i.e., over-increasing the phylogenetic signal) were high. To verify these risks, we plotted onto the Springer’s phylogeny^71^ the genital bone occurrence reported in Brindle and Opie^28^ and compared it with our state of knowledge (Fig. 1) as shown in Supplementary Fig. S8. Expectedly, there was incomplete concordance between their and our *baculum* occurrence data at both the genus and the species levels. For example, Brindle and Opie^28^ reported two absences at the genus level in *Lagothrix* and *Alouatta*, while we found four presences at the species level within those genera, such as: *L. lagothricha*^62^*, A. caraya* (Humboldt, 1812)^62^, *A. guariba* (Humboldt, 1812)^9^, and *A. seniculus* (Linnaeus, 1766) (one of our original scanned specimens, American Museum of Natural History [AMNH] wet primate collection). Similarly, Brindle and Opie^28^ reported a presence in both *Pithecia* and *Pygathrix* genus, while we found one absence in *P. irrorata*^19^ and in *P. nigripes* (3 scanned adult penises). In conclusion, the interspecific variation affecting both occurrence and morphology of mammal genital bones^25,38,43,48,65,85,86^ would definitively be flattened by the a priori hypothesis that congeneric species share *baculum* state (see also^26^). That hypothesis should therefore be rejected. Nevertheless, the wide distribution of the *baculum* throughout the entire primate order, as truly thoroughly demonstrated by our occurrence dataset, brought Brindle and Opie^28^ to a correct conclusion as well.

The sample size is known to give analytical strength, and the overall incomplete data availability for genital bone occurrence has certainly represented an obstacle in primates, but especially so for the study of the *baubellum*. For example, in the description of both male and female genital anatomy made by Hill in one of the widest anatomical treatises on primates^15^, the frequency of female genital dissections and searches for a genital bone did not equal the males’. Surprisingly, in most studies on external female genitals (though including dissections) either the presence or the absence of a *baubellum* was omitted as well (e.g.^6,7,21,60^). Several other authors incurred such omissions, generating most of the ‘omitted data’ about this topic (e.g.,^17,18,87^). Also, and as a consequence of this, the paucity of *baubellum* occurrence data rigorously matched with the absence of *baubellum* functional hypotheses and adaptive meanings in the literature. Interestingly, a substantial male sex bias also persisted in live-collected and subfossil mammal museum collections, with males outnumbering females in primate collections as well. A range of plausible factors which might have been facilitating a biased sampling focused on males in the history of field expeditions could be those contributing to higher male visibility and detectability due to sexual dimorphisms, in terms of (1) behaviour, (2) body mass, and (3) wider male geographic ranges (mainly increasing chances of detection of fossils)^88,89^.

If compared to the thus far available dataset on *baubellum* occurrence in primate species, our dataset almost triplicated the number of species (27 species in^29^, 78 species in this study). Namely, new reports of occurrence in the present study were in the Hylobatidae fam. (*Hylobates* clade, and *Hoolock* clade), the Cebidae fam. (*Sapajus* clade), the Callitrichidae fam. (*Callithrix* clade), and several species in sparse nodes of strepsirrhines. We also differed in some data interpretation as those reported as presence even if based on cartilage, such as the case of *baubellum* presence in *Loris tardigradus* (Linnaeus, 1758)^62^, and the case of polymorphism (i.e., absence-presence within a species) in *Galago senegalensis* É. Geoffroy Saint-Hilaire, 1796^60,61^.

We nevertheless have to highlight that some of the incongruencies in the occurrence data found at both the inter- and intra-specific levels, might indeed reflect an actual variability affecting both genital bones. Thanks to our methodological approach (micro-CT scan) that conferred high reliability for absence data, we were able to establish, for the first time, the existence of variability in the occurrence of both *baculum* and *baubellum* at the intra-specific level (as recently presumed for *baculum* by Jakovlić^90^). For example, by comparing scanned versus literature occurrence data we found: *baculum* absence versus presence in 14 species; *baculum* presence versus absence in 1 species (i.e., one more species with *baculum*); *baubellum* absence versus presence in 6 species; *baubellum* presence versus absence never occurred. Even when limited to scanned samples only, we also found either presence or absence of *baculum* in several specimens belonging to the same species, that are: *Callithrix jacchus* (Linnaeus, 1758), *Cercopithecus albogularis* Sykes, 1831, *Chlorocebus pygerythrus* (Cuvier, 1821), *Colobus guereza* Rüppel, 1835, *Lemur catta* Linnaeus 1758, *Macaca fascicularis* (Raffles, 1821), *Macaca fuscata* (Blyth, 1875), *Macaca mulatta* (Zimmermann, 1780), *Otolemur garnettii* (Ogilby, 1838), *Pan troglodytes* (Blumenbach, 1775), *Papio hamadryas* (Linnaeus, 1758), *Papio ursinus* (Kerr, 1792).

In our study, all museum and fresh samples have undergone the same analytical investigation^91^ with no exceptions. We acknowledge that although micro-CT scan is a well-known reliable technique to detect bones inside soft tissues (i.e., bones usually totally radiopaque to X-rays), the actual presence of genital bones have traditionally derived (but see^92^) from direct pieces of evidence (i.e., invasive dissection and/or histochemistry). However, since all our doubtful cases were limited to museum samples, invasive techniques were not allowed.

Although based on a much larger dataset, our analysis could not clarify whether the common primate ancestor possessed a *baubellum* (i.e., the posterior probability was 52%). In particular, while the ancestor of strepsirrhines could have perhaps possessed a clitoral bone (73% of posterior probability), the heterogeneity of haplorrhine data (31 absences and 12 presences), did not allow the detection of a clear pattern of *baubellum* evolution for this clade (54%). In contrast, the analysis performed by Lough-Stevens et al.^29^ seemed to strongly support *baubellum* ancestry in the primate order (although not discussed in the study; but see primate node colour of their Fig. 2). The unresolved ancestry based on our bigger dataset compared to the apparent clear output based on their smaller dataset was, at the very least, unexpected. Nevertheless, a possible explanation perhaps lies in the higher proportion of absences compared to the total amount of data in this study, that is 51% (i.e., 37 absences out of 72 data points) *versus* the lower proportion in Lough-Stevens et al.^29^, that is 17% (i.e., four absences out of 23 data points).

In agreement with Lough-Stevens et al.^29^ (see also^25,65,93^), we confirmed the univocal pattern of *baubellum* presence consistently associated with *baculum* presence at the species level (but not the opposite). Genital bones did share high levels of state correlation, that is they were either both present or absent in 44 out of the 71 species investigated (62%). Indeed, this correspondence pattern was not unexpected considering the well-established homology between *baculum* and *baubellum* (see for primates^18,19,25,57,64,65^; for non-primates^66,67^). Interestingly, and similar to *baculum*, the results of analyses for *baubellum* seem largely independent of the phylogeny here used.

The concept of homology, at this point, needs to be deepened also considering the results of the most recent publications about genital bones in mammals questioning about it (for *baculum*^26^; for *baubellum*^29^). They reported that since the *baculum* has evolved independently 9 times in mammals (two of which in primates), then the assumption of homology was violated. According to the most used terminology (reviewed in^94^), the non-homology should reflect either “parallelism” or “convergent evolution”, raising the question of whether the *baculum* might have arisen via different genetic and developmental pathways^95,96^. Conversely, both parallelism and convergent evolution could be considered as two sides of the same coin, that is homoplasy. Homoplasy and homology could be intended as the extremes of a continuum “reflecting deep or more recent shared ancestry based on shared cellular mechanisms and processes and shared genes and gene pathways and networks”^97^. Based on this, if the rationale of Schultz et al.^26^ and of Lough-Stevens et al.^29^ was valid and applied to *baculum* and *baubellum* evolution in primates, we could not easily explain the following facts: (a) the *baculum* is always placed inside the penis and always surrounded by the same tissues^9,15,81,98^, (b) the *baubellum* (when present) is always placed inside the clitoris (homologous to the penis) and always associated to the *baculum* intra-species^28,29^ (this study), and (c) *baculum* and *baubellum* share the same regulatory mechanism of gene expression during development^66,67^. On the contrary, if we start from these well-known pieces of evidence, and despite the data available for *baubellum* so far are still inadequate to reflect its evolutionary history across the primate order (especially in haplorrhines), we can re-interpret our *baubellum* ancestry output. With this view, we ultimately propose that the scattered absences of *baubellum* in the haplorrhines are, indeed, losses. In this perspective, *baubellum* evolution might well agree with primate *baculum* ancestry^28^ (this study).

This study provided the most up-to-date and solid evidence contributing to address the evolution of genital bones in primates. With this study, we aimed at encouraging future research to put more effort into the generation of databases as reliable as updated. For the time being, it is important to both acquire additional occurrence data by exploring primate museum collections and apply the most rigorous methodological protocol available for detecting absences with as much certainty as possible. In addition, updated distribution of *ossa genitalia* in primates (also supported by more comprehensive primate phylogenies), as well as comparative morphometric studies (e.g.,^99^), would allow to better understand both their evolutionary history and adaptive function in a sexual selection framework.

## Methods

The present work aimed at (i) updating the dataset resulting from both literature search and new records of primate genital bones (by the sampling of both fresh and museum specimens); (ii) establish a renewed/robust starting point for further studies about the evolution of these bones by offering an exhaustive dataset of the genital bone occurrence in primates at the species level; (iii) performing the genital bone ancestral state reconstruction within the primate clade, based on the largest and updated dataset currently available.

### The primary literature search

The primary literature considered in this work consisted only of papers, books and texts reporting explicitly and for the first time either the presence or the absence of genital bones in a primate specimen. To collect data about the occurrence of both *baculum* and *baubellum*, our literature search followed some significant ‘steps’. We applied the ‘berry picking’ model for information retrieval^100^ which started from a general query to make the user able, by examining research results, to easily identify specific teams working on this topic. Then, by selecting a few papers that answered (totally or partially) the main query, six specific tactics had to be followed:

1. *backward chaining* or footnote chasing, by following references (and footnotes) in books and articles of interest, and moving backwards through a chain of the reference list;
2. *forward chaining* or citation searching, by starting with a citation, finding out who cites it, and following the chain in a forward direction;
3. *journal run*, hand searching relevant journals;
4. *area scanning*, browsing materials physically collocated and accessible;
5. *subject searches*, in bibliographies and abstracting and indexing (A & I) services, considering that many bibliographies and most A & I services are arranged by subject;
6. *author searching*, to understand if the author has done any other work on the same topic.

Furthermore, we also added the reading of anatomy papers, treatises, and books, which described primate external genitals, even if not resulted from the web search. By following this protocol, we were able to browse publications ranging from 1871 to 2012 (for a total of N = 117 references) to generate our occurrence dataset for both *baculum* and *baubellum* named Supplementary Table S1. It is important to stress that Supplementary Table S1 strictly reflects the literature investigated. How we treated specific taxonomic and uncertainty issues is explained in detail in the subsequent paragraphs.

### Genital bone sampling

We collected 148 specimens as a whole (either genital samples N = 45, or entire corpses, N = 103) for 68 primate species and 2 subspecies, as well as a few specimens identified only at the genus level (i.e., 3 primate genera) (Supplementary Table S2). Samples were of two kinds: (1) fresh samples (N = 16; 9 clitorises and 7 penises) and (2) museum wet samples (N = 132; 1 *baculum*, 23 clitorises, 92 penises, 8 female whole bodies, 8 male whole bodies). Fresh samples were obtained from fresh cadavers of primate specimens, dead for natural causes, before necropsy investigations (in collaboration with Italian Istituti Zooprofilattici Sperimentali, see^91^ for further details). Museum wet samples were obtained from (i) the «Museo di Zoologia dell’Università di Torino» (MZUTT, Turin, IT, theriological collection^101^; N = 1); (ii) the Natural History Museum «La Specola», (NHMLS, Zoological Section, Florence, IT, non-human primate collection^102^; N = 1); (iii) the American Museum of Natural History (AMNH, New York, USA, Vertebrate Collection Database available at http://sci-web-001.amnh.org/db/emuwebamnh/index.php; N = 46); (iv) the National Museum of Natural History (NMNH, Washington, D.C., USA, Mammal Collection Database available at https://collections.nmnh.si.edu/search/mammals/; N = 84).

In order to obtain data about the genital bone either presence or absence, the 3-step methodological protocol proposed by Spani et al.^91^ was applied (i.e., palpation method, X-rays, and micro-CT scanner). Three different micro-CT scanners have been used depending on the location of collected samples, whether Italy (same used by Spani et al.^91^) or USA. (PHOENIX V|TOME|X S, available at AMNH, and PHOENIX V|TOME|X M, available at NMNH) and machine settings have been reported in Table 2.

**Table 2:** Micro-CT setups.

Parameter	Setting (ITA)	Setting (USA)
Voltage (kV)	60–100	Auto
Beam current (µA)	80–200	Auto
Al filter (mm)	None/1	None/2
No projections	900	1500–1800
Total rotation angle	360°	360°
Exposure time (s)	0.7–1	1
Voxel size (µm)	9.16–14	18

Micro-Computed Tomography setups used for scanning both Italian and American specimens of primate external genitals (auto = set automatically).

### Taxonomical issues

The increasing number of primate species since the 1990s was the result of the descriptions of newly discovered species and subspecies, as well as taxonomic revisions, molecular studies and growing usage of the Phylogenetic Species Concepts^103,104^. Most old anatomical treatises were based on a primate taxonomy which sometimes did not match with the current taxonomy^7–14,81^. Taxonomical issues might increase the potential of wrong occurrence data assignments as when a species has been split into two species, or a subspecies has been elevated to species level, or simply species synonyms were used. To overcome this potential source of confusion, we relied on two well established taxonomical databases that offer standardized nomenclature to create easily accessible and reliable information on species names and their hierarchical classification: (i) IUCN Red List on-line database (467 extant primate species, as of the year 2019^70^) (ii); ITIS on-line database (508 extant primate species, as of the year 2020^84^). The IUCN Red List database^70^ was our primary source consulted to disentangle taxonomical incongruences and to reconstruct the species-specific nomenclature history. Double checks were done by searching controversial species names also on ITIS database^84^. Both the original and current names have been reported into the ‘Notes’ column of Supplementary Table S1. Once the correct link between old and current nomenclature at the specific level was created, we chose the following criteria to adapt the original data to the present nomenclature, trying to avoid data loss: (a) current species derived from the split of the original species found in old literature, were given the same occurrence data as in the original species; (b) subspecies of the original species found in old literature, now ‘upgraded’ to the species level, were given the same occurrence data as in the original subspecies; (c) junior synonyms of the original species found in old literature were given the same occurrence data as in the original earlier synonym. These cases have been labeled as ‘inferred’ in the ‘References’ column in Supplementary Table S1 and accounted for 36.9% of all species for *baculum* presence/absence data, and 36.2% for *baubellum* presence/absence data.

### Doubtful cases of occurrence

During the generation of our data matrix, we tackled a variety of doubtful cases of occurrence, which overall might be summarized around three main kinds.

Some authors^11,18,57,82,105^ attributed presence/absence data to the genus simply relying on observations made on one or a few specimens, whose species name, however, was not mentioned in the text. In such cases, we first checked if any text figures, showing genital sections, included any species name in the figure caption. If so, we assigned presence/absence data to that species. On the contrary, if neither figure legends were helpful, nor more recent references for one/more species belonging to that genus were available, the *datum* of occurrence was recorded in the column ‘P/A gen’, relative to genus level, but not in the column ‘P/A sp’, relative to species level. It is important to note that column ‘P/A gen’ of Supplementary Table S1 was not included in the data matrix used for the reconstruction of the ancestral state of the character (see “The presence/absence binary matrix and the trait transition analysis ” paragraph below), however, was recorded in the table for completeness of data.

We also handled conflicting information found in the literature about a certain degree of supposed intraspecific/interindividual variability in the occurrence of *ossa genitalia* associated with different mentions as to whether the structure found was either bone or cartilage. For example, Hill^83^ reported that the *baculum* is absent in *L. lagothricha*, while Machida and Giacometti^62^ reported the presence of a “short rod bone or cartilage”. We found similar cases regarding the *baubellum*: (i) *G. senegalensis* was reported by Petter-Rousseaux^60^ as lacking it, while Butler^61^ reported the presence of a “cartilaginous clitoral bone”; (ii) *L. tardigradus* was reported by Hill^54^ as lacking it, while Machida and Giacometti^62^ reported the presence of a “cartilaginous *baubellum*”; (iii) *Ateles paniscus* (= *Ateles ater*) (Linnaeus, 1758) was reported by Pehrson^10^ as lacking it (dissection), while later Harms^16^ generically referred to whole genus *Ateles* as having a “clitoral cartilage” (but the only species mentioned in a figure caption was *A. belzebuth* É. Geoffroy, 1806), followed by Ioannou^17^ referring to a generic ‘*Ateles’* and stating “a *baculum* [read *baubellum*] may be present”; (iv) *Hoolock hoolock* (Harlan, 1834) was reported by Matthews^56^ as lacking it, while Geissmann and Lim^21^ found a very small *os clitoridis* in one female specimen of *H. hoolock*. All conflicting results are reported as such in the synthetic Supplementary Table S1. For the analyses, however, we only considered the most conservative data, i.e., the explicit mention of a bone only at the species level (*baculum* was set as present in *L. lagotricha* and *baubellum* was set as present in *H. hoolock*), discarding cartilaginous structures (*baubellum* was set as absent in *G. senegalensis*, *L. tardigradus*, *A. belzebuth*, and *A. paniscus*).

**Figure 5:**
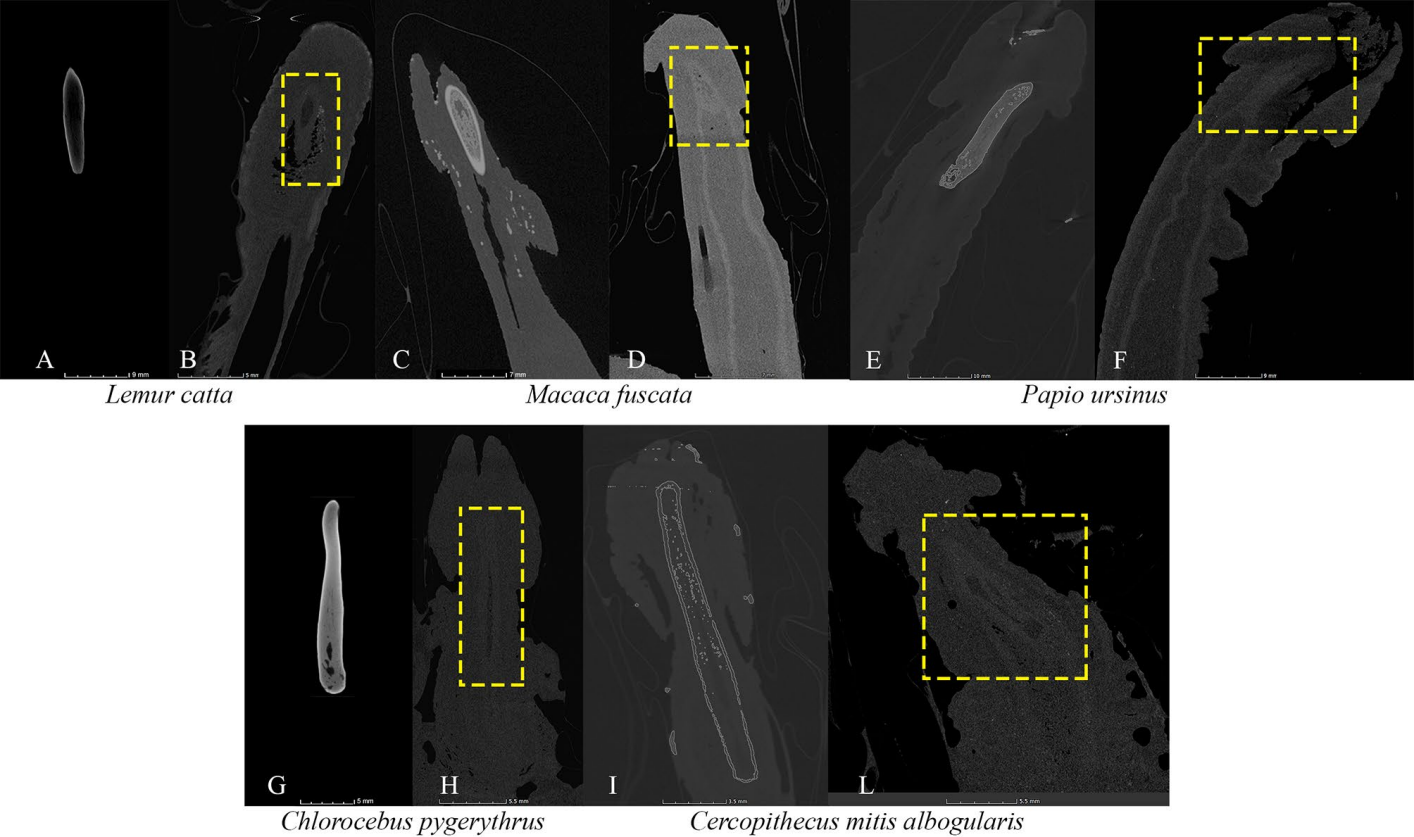
Doubtful cases of *baculum* occurrence in micro-CT scanned samples. Reconstructed micro-CT slices of doubtful cases of *baculum* occurrence: specimens showing a *baculum* on the left (**A,C,E,G,I**); in specimens (**A,G**) the penile bone was extracted from soft tissues) compared to conspecific doubtful cases highlighted with dashed yellow lines on the right (**B,D,F,H,L**). Not to scale. Figure generated in myVGL (v. 3.0, https:// https://www.volumegraphics.com/).

The last kind of doubtful cases of genital bone occurrence emerged from micro-CT scanned samples (for methodological details please see^91^). We classified these cases as ‘doubtful’ (noted with ‘?’ in Supplementary Table S2, columns ‘Present study data—micro-CT’) because reconstructed slices showed a ‘shadow’ (where the *baculum* is usually placed) characterized by grey levels which were intermediate between those typical of a bone absence and those typical of a presence. Figure 5 showed some examples of doubtful cases and compared reconstructed CT slices belonging to two individuals of the same species (1 presence vs. 1 doubtful occurrence). In these cases, for the trait transition analyses, we considered the character present in a species when either documented in the literature or resulting from CT-scanned samples.

### Ancestral character state analysis: occurrence binary matrix and stochastic character mapping

Based on both Supplementary Tables S1 and S2, a presence/absence database on the occurrence of *baculum* and *baubellum* in primate species was generated by using a binary code (1 = presence, 0 = absence) for analysis. The binary database ready for analytical purposes differed from exhaustive Supplementary Tables S1 and S2, as it only included species-specific “data *certa*”, i.e., never attributing the genus datum to corresponding congeneric species (therefore risking data inflating), and never swapping out data between species. We chose a robust molecular phylogeny that included 70 primate genera and 367 primate species based on a concatenation of 69 nuclear gene segments (54 of them were taken from Perelman et al.’s^68^ nexus file with some modifications) and ten mitochondrial gene sequences (see^71^ for more details about chosen primate phylogeny). This was the most complete and robust available phylogeny to our knowledge. However, neither all species appearing in the phylogeny (N = 367) had an occurrence datum, nor all species with genital bone data (N = 280 for *baculum*, and N = 78 for *baubellum*, as a total deriving from the sum of both literature and scan data) were included in the reconstructed molecular phylogeny, therefore we limited the analyses to the species included in the phylogeny by finally using binary matrixes of N = 244 species for *baculum* and N = 72 species for *baubellum*. Once data were mapped onto the Springer’s primate phylogeny^70^, the analyses were performed separately for both *baculum* and *baubellum.* To unlink our investigation from the tree topology of Springer et al., we repeated the analysis using the primates tree as provided by the Timetree resource^72^. Timetree provided an ultrametric, quasi-fully resolved tree with 364 species. The tree was based on 52 molecular phylogeny studies of primates and polytomies were resolved as described in Hedges et al.^106^. The pruning procedure that we applied on the Timetree phylogeny allowed us to retain 224 of the original 364 species in the tree.

We investigated the trait evolution using the stochastic character mapping method^107,108^. The method allows sampling possible histories of a discrete character state from their Bayesian posterior probability distribution. Using a large number of maps it is possible to use the variability among sampled histories to account for the uncertainty about trait evolution across a phylogeny. The method has also the desirable property to accommodate uncertainty in the phylogeny of the group when mapping characters. The stochastic maps are constrained to be consistent with the observed character states. Because the stochastic character mapping analysis results in a large number of discrete character histories on a phylogeny, proper visualization may be obtained by applying Method 1 presented by Revell^109^. This is a technique to visualize the posterior density of character histories obtained by aggregating the results from a set of stochastic character maps. We first used the function make.simmap wth argument Q = ‘mcmc’, of the phylogenetic R package ‘phytools’^110^ to run 1000 iterations of stochastic mapping (i.e., 1000 trees were built with the same character state on tips as observed in the input tree, but with different histories). The process is regulated by a rate matrix that carries the information to calculate the transition probabilities. These are the probability that the process of transition along a branch ends in a certain state after a certain number of transitions, having started from a different state. In this procedure, each branch of the tree was fractioned and the posterior probability of the character state being 0 (absent) or 1 (present) was computed, for each fraction of the branch, as the relative frequency across all stochastic maps. Visualization was obtained using the function densityMap of the phylogenetic R package ‘phytools’^110^. In this way, it was possible to plot the cumulative probability of the character state transition on the tree branches by using a colour map for translating the probability to a plotted colour. The result was a continuous colour changing along branches of the tree that expressed the posterior probability. The trait transition analyses were run for both *baculum* ad *baubellum*. We first ran an analysis by including five outgroups^71^ whose data of genital bone occurrence were derived from Schultz et al.^26^ for *baculum* and Lough-Stevens et al.^29^ for *baubellum*. Subsequently, to evaluate to what extent the condition of the character state in the outgroups could influence the trait transition across primates, we repeated the analyses without the five outgroups.

Crucial for the analysis of trait evolution is the selection of the proper evolutionary model for the matrix Q that describes the rates of transition between states. The All-Rates-Different and Symmetrical models (ARD and SYM, respectively^111^) were tested using the function pchisq in the R package ‘stats’. The two models for discrete character evolution are specific cases of the Mk model^112^ which applies to a discrete character having ‘k’ unordered states and involves state transition between these k states. In the SYM model, the rate of change between any two-character states is the same forwards as it is backward. The ARD model allows every possible type of transition to have a different rate. The choice of using the SYM model was based on the results of the likelihood test conducted on *baculum* and *baubellum*. In fact, for *baculum*, the ARD model was only marginally preferred over the SYM model, as the twice-the-difference in likelihoods (4.306_Springer_; 5.376_Timetree_; 1df) between the two models laid in the largest 5% of values rightmost tail of the χ^2^ distribution, but not in the most conservative largest 1%. For *baubellum*, the SYM model was preferred over the ARD, as the twice-the-difference in likelihoods (0.071_Springer_; 0.007_Timetree_; 1df), was not statistically significant.

Following an independent approach, we investigated also the level of character dispersion on the phylogenetic tree (phylogenetic signal strength) by estimating the D statistic^113^. The statistic is based on the sum of sister-clades differences in terms of the presence/absence of a specific trait (character state). The D statistic results in a higher value if the character state is overdispersed across a phylogeny, whereas it shows lower values when the character state is strongly clumped.

## Supplementary Information

Supplementary Legends.

Supplementary Table S1.

Supplementary Table S2.

Supplementary Figure S3.

Supplementary Table S4.

Supplementary Figure S5.

Supplementary Figure S6.

Supplementary Figure S7.

Supplementary Figure S8.

## Data Availability

All data generated during this study are included in this published article [and its supplementary information files]. The datasets analyzed during the current study are available from the corresponding author on reasonable request.

## References

[1] Brocke, vom J.*et al*. Reconstructing the giant: On the importance of rigour in documenting the literature search process. In *ECIS 2009 Proceedings* 161 (2009).

[2] Newton, I. & Hooke, R. *Isaac Newton letter to Robert Hooke* (1675).

[3] Weatherall DJ (2013). On the shoulders of giants. The Lancet.

[4] vom Brocke J (2015). Standing on the shoulders of giants: Challenges and recommendations of literature search in information systems research. CAIS.

[5] Grandidier A (1871). Observations sur les Propitheques de Madagascar. C. R. Acad. Sci..

[6] Harcourt AH, Gardiner J (1994). Sexual selection and genital anatomy of male primates. Proc. R. Soc. B.

[7] Gerhardt U (1909). Ueber das Vorkommen eines Penis-und Clitorisknochens bei Hylobatiden. Ann. Anat..

[8] von Pehrson T (1910). Beitrage zur Kenntnis des Os penis der Prosimier. Ann. Anat..

[9] von Pehrson T (1928). Zur Morphologie der männlichen Kopulationsorgane der Säugetiere; insbesondere der Versuch einer vergleichend-anatomischen Studie über den Penis der Primaten, einschließlich des Menschen. Brain Struct. Funct..

[10] von Pehrson T (1914). Beiträge zur Kenntnis der äusseren weiblichen Genitalien bei Affen, Halbaffen, und Insectivoren. Ann. Anat..

[11] Pocock RI (1918). On the external characters of the Lemurs and of Tarsius. J Zool..

[12] Pocock RI (1920). On the external characters of the South American Monkeys. J Zool..

[13] Pocock RI (1925). The external characters of the Catarrhine Monkeys and Apes. J Zool..

[14] Wislocki GB (1936). The external genitalia of the simian primates. Hum. Biol..

[15] Hill, O. W. C. *Primates-Comparative Anatomy and Taxonomy* (University Press, 1953–1974).

[16] Harms JW, Hofer H, Schultz AH, Starck D (1956). Volume I: Systematik. Phylogenie. Ontogenie. Primatologia. Handbuch der Primatenkunde.

[17] Ioannou JM, Hafex ESE (1971). Vol. 6: Female reproductive organs. Comparative Reproduction of Nonhuman Primates.

[18] Hershkovitz P, Hershkovitz P (1977). Vol. 1(2): 14. External genitalia and accessory structures. Living New World Monkeys (Platyrrhini) with an Introduction to Primates.

[19] Hershkovitz P (1993). Male external genitalia of non-prehensile tailed South-American monkeys. Part I. Subfamily Pitheciinae, Family Cebidae. Fieldiana Zool..

[20] Dixson AF (1987). Baculum length and copulatory behavior in primates. Am. J. Primatol..

[21] Geissmann T, Lim KKP (1994). Extraction of bacula of tanned gibbons skins. Raffles Bull. Zool..

[22] Anderson MJ (2000). Penile morphology and classification of bush babies (subfamily Galagoninae). Int. J. Primatol..

[23] Carosi M, Ulland AE, Gerald MS, Suomi SJ (2001). Male-like external genitalia in female tufted capuchins (*Cebus apella*), and the presence of a clitoral bone (baubellum): A cross-sectional study. Folia Primatol..

[24] Dixson AF, Pissinatti A, Anderson MJ (2004). Observations on genital morphology and anatomy of a hybrid male muriqui (genus Brachyteles). Folia Primatol..

[25] Dixson AF (2012). Primate Sexuality: Comparative Studies of the Prosimians, Monkeys, Apes, and Humans.

[26] Schultz NG, Lough-Stevens M, Abreu E, Orr T, Dean MD (2016). The Baculum was gained and lost multiple times during mammalian evolution. Integr. Comp. Biol..

[27] Orr TJ, Brennan PL (2016). All features great and small—The potential roles of the baculum and penile spines in mammals. Integr. Comp. Biol..

[28] Brindle M, Opie C (2016). Postcopulatory sexual selection influences baculum evolution in primates and carnivores. Proc. R. Soc. B.

[29] Lough-Stevens M, Schultz NG, Dean MD (2018). The baubellum is more developmentally and evolutionarily labile than the baculum. Ecol. Evol..

[30] Dixson AF, Anderson M (2001). Sexual selection and the comparative anatomy of reproduction in monkeys, apes, and human beings. Annu. Rev. Sex Res..

[31] Dixson AF (1987). Observations on the evolution of the genitalia and copulatory behaviour in male primates. J. Zool..

[32] Dixson AF (1995). Baculum length and copulatory behaviour in carnivores and pinnipeds (Grand Order Ferae). J. Zool..

[33] Miller EH, Jones IL, Stenson GB (1999). Baculum and testes of the hooded seal (*Cystophora cristata*): Growth and size-scaling and their relationships to sexual selection. Can. J. Zool..

[34] Hosken D, Jones K, Chipperfield K, Dixson A (2001). Is the bat os penis sexually selected?. Behav. Ecol. Sociobiol..

[35] Lariviére S, Ferguson SH (2002). On the evolution of the mammalian baculum: Vaginal friction, prolonged intromission or induced ovulation?. Mamm. Rev..

[36] Miller EH, Burton LE (2001). It’s all relative: Allometry and variation in the baculum (os penis) of the harp seal, *Pagophilus groenlandicus* (Carnivora: Phocidae). Biol. J. Linn. Soc..

[37] Lüpold S, McElligott AG, Hosken DJ (2004). Bat genitalia: Allometry, variation and good genes. Biol. J. Linn. Soc..

[38] Ramm SA (2007). Sexual selection and genital evolution in mammals: A phylogenetic analysis of baculum length. Am. Nat..

[39] Tasikas D, Fairn E, Laurence S, Schulte-Hostedde A (2009). Baculum variation and allometry in the muskrat (*Ondatra zibethicus*): A case for sexual selection. Evol. Ecol..

[40] Ramm S, Khoo L, Stockley P (2010). Sexual selection and the rodent baculum: An intraspecific study in the house mouse (*Mus musculus domesticus*). Genetica.

[41] Schulte-Hostedde AI, Bowman J, Middel KR (2011). Allometry of the baculum and sexual size dimorphism in American martens and fishers (Mammalia: Mustelidae). Biol. J. Linn. Soc..

[42] Fitzpatrick JL, Almbro M, Gonzalez-Voyer A, Kolm N, Simmons LW (2012). Male contest competition and the coevolution of weaponry and testes in pinnipeds. Evolution.

[43] Simmons LW, Firman RC (2013). Experimental evidence for the evolution of the mammalian baculum by sexual selection. Evolution.

[44] Stockley P (2013). Baculum morphology predicts reproductive success of male house mice under sexual selection. BMC Biol..

[45] Simokawa S (1938). Einige Bemerkungen fiber den Clitorisknochen Keijo. J. Med..

[46] Jellison WL (1945). A suggested homolog of the os penis or baculum of mammals. J. Mammal..

[47] Layne JN (1954). The os clitoridis of some North American Sciuridae. J. Mamm..

[48] Burt WH (1936). A study of the baculum in the genera *Perognathus* and *Dipodomys*. J. Mammal..

[49] Brown RE (1967). Bacula of some new world molossid bats. Mammalia.

[50] Brown RE, Genoways HH, Jones JJK (1971). Bacula of some Neotropical bats. Mammalia.

[51] Sutton DA (1982). The female genital bone of chipmunks, genus *Eutamias*. Southwestern Nat..

[52] Carosi M, Spani F, Ulland AE, Scalici M, Suomi SJ (2020). Clitoral length in immature and mature captive tufted capuchin (*Sapajus* spp.) females: A cross-sectional study. Am J Primatol..

[53] Rau AS, Hiriyannaiya S (1930). Contributions to our knowledge of the anatomy of the Lemuroidea. II. The urinogenital system of Loris lydekkerianus. J. Mysore Univ..

[54] Hill OWC (1933). A monograph on the Genus *Loris*: With an account of the external, cranial and dental characters of the genus: A revision of the known forms, and the description of a new form from northern Ceylon. Ceylon J. Sci..

[55] Clark WEG (1934). Early Forerunners of Man.

[56] Matthews LH (1946). Notes on the genital anatomy and physiology of the gibbon (Hylobates). J. Zool..

[57] Hill OWC (1953). Primates-Comparative Anatomy and Taxonomy.

[58] Hill OWC, Davies DV (1954). XVII-The reproductive organs in *Hapalemur* and *Lepilemur*. Proc. R. Soc. Edinb. B..

[59] Hill OWCB, Hofer H, Schultz AH, Starck D (1958). Vol. 3: External genitalia. Primatologia.

[60] Petter-Rousseaux A (1962). Recherches sur la biologie de la reproduction de Primates inférieurs. Mammalia.

[61] Butler H (1964). The reproductive biology of a strepsirrhine (*Galago senegalensis senegalensis*). Int. Rev. Gen. Exp. Zool..

[62] Machida H, Giacometti L (1967). The anatomical and histochemical properties on the skin of the external genitalia of the primates. Folia Primatol..

[63] Groves CP, Rumbaught DM (1972). Vol. 1: Systematics and phylogeny of gibbons. Gibbons and Siamang.

[64] Hill OWC (1972). Evolutionary Biology of Primates.

[65] Stockley P (2012). The baculum. Curr. Biol..

[66] Glucksmann A, Cherry CP (1972). The hormonal induction of an os clitoridis in the neonatal and adult rat. J. Anat..

[67] Murakami R (1987). A histological study of the development of the penis of wild-type and androgen-insensitive mice. J. Anat..

[68] Perelman P (2011). A molecular phylogeny of living primates. PLoS Genet..

[69] Arnold C, Matthews LJ, Nunn CL (2010). The 10KTrees website: A new online resource for primate phylogeny. Evol. Anthropol..

[70] IUCN. *The IUCN Red List of Threarened Species. Version 2019-3* (2019). http://www.iucnredlist.org (Accessed February, 25th 2019).

[71] Springer MS (2012). Macroevolutionary dynamics and historical biogeography of primate diversification inferred from a species supermatrix. PLoS ONE.

[72] Kumar S, Stecher G, Suleski M, Hedges SB (2017). TimeTree: A resource for timelines, timetrees, and divergence times. Mol. Biol. Evol..

[73] Bett WR (1951). The os penis in man and beast. J. R. Soc. Med..

[74] Bett WR (1952). The os penis in man and beast. Ann. R. Coll. Surg. Engl..

[75] Eglitis JA (1953). Occurrence of bone tissue in the human penis. J. Urol..

[76] Sarma DP, Weilbaecher TG (1990). Human os penis. Urology.

[77] Champion RH, Wegrzyn J (1964). Congenital *Os penis*. J. Urol..

[78] Ruth EB (1934). The os priapi: A study in bone development. Anat. Rec..

[79] Napier JR, Napier PH (1985). The Natural History of the Primates.

[80] Dixson A (1998). Primate Sexuality: Comparative Studies of the Prosimians, Monkeys, Apes, and Human Beings.

[81] Woollard HH (1925). The anatomy of *Tarsius spectrum*. J. Zool..

[82] Hill OWC (1955). Primates—Comparative Anatomy and Taxonomy.

[83] Hill OWC (1953). Observations on the genitalia of the Woolly Monkey (*Lagothrix*). J. Zool..

[84] ITIS. *Integrated Taxonomic Information System. Version 2020* (2020). http://www.itis.gov (Accessed February 24, 2020).

[85] Burt WH (1960). Bacula of north american mammals.

[86] Patterson BD, Thaeler CS (1982). The mammalian baculum: Hypotheses on the nature of bacular variability. J. Mammal..

[87] Wilson MI (1977). A note on the external genitalia of female squirrel monkeys (*Saimiri sciureus*). J. Med. Primatol..

[88] Cooper N (2019). Sex biases in bird and mammal natural history collections. Proc. R. Soc. B.

[89] Gower G (2019). Widespread male sex bias in mammal fossil and museum collections. Proc. Natl. Acad. Sci. U.S.A..

[90] Jakovlić I (2021). The missing human baculum: A victim of conspecific aggression and budding self-awareness?. Mamm. Rev..

[91] Spani F, Morigi MP, Bettuzzi M, Scalici M, Carosi M (2020). A 3D journey on virtual surfaces and inner structure of *Ossa genitalia* in Primates by means of a non-invasive imaging tool. PLoS ONE.

[92] Brassey CA, Gardiner JD, Kitchener AC (2018). Testing hypotheses for the function of the carnivoran baculum using finite-element analysis. Proc. R. Soc. B.

[93] Carosi M, Scalici M, Linn GS, Fuentes A, Bezanson M, Campbell CJ (2016). Baubellum (*Os clitoridis*). The International Encyclopedia of Primatology.

[94] Arendt J, Reznick D (2008). Convergence and parallelism reconsidered: What have we learned about the genetics of adaptation?. Trends Ecol. Evol..

[95] Haldane JBS (1932). The Causes of Evolution.

[96] Simpson GG (1952). The Meaning of Evolution.

[97] Hall BK (2007). Homoplasy and homology: Dichotomy or continuum?. J. Hum. Evol..

[98] Carosi M, Scalici M, Fuentes A, Bezanson M, Campbell CJ (2016). Baculum (*Os Penis*). The International Encyclopedia of Primatology.

[99] Brassey CA, Behnsen J, Gardiner JD (2020). Postcopulatory sexual selection and the evolution of shape complexity in the carnivoran baculum. Proc. R. Soc. B..

[100] Bates MJ (1989). The design of browsing and berry-picking techniques for the online search interface. Online Rev..

[101] Calvini M, Siori MS, Gippoliti S, Pavia M (2016). Catalogue of the primatological collection of the Torino University. Nat. Hist. Sci..

[102] Veracini C, Ducci L, Agnelli P (2010). Review and historical notes on the non human primate collection of the Natural History Museum, Zoological Section «La Specola», Florence University, Italy. Atti Soc. Tosc. Sc. Nat..

[103] Cracraft J, Johnston RF (1983). Vl 1: Species concepts and speciation analysis. Current Ornithology.

[104] Rylands AB, Mittermeier RA (2014). Primate taxonomy: Species and conservation. Evol. Anthropol..

[105] Harrison RM, Lewis RW, Dukelow WR, Erwin J (1986). Vol. 3: The male reproductive tract and its fluids. Comparative Primate Biology.

[106] Hedges SB, Marin J, Suleski M, Paymer M, Kumar S (2015). Tree of life reveals clock-like speciation and diversification. Mol. Biol. Evol..

[107] Nielsen R (2002). Mapping mutations on phylogenies. Syst. Biol..

[108] Huelsenbeck JP, Nielsen R, Bollback JP (2003). Stochastic mapping of morphological characters. Syst. Biol..

[109] Revell LJ (2013). Two new graphical methods for mapping trait evolution on phylogenies. Methods Ecol. Evol..

[110] Revell LJ (2012). phytools: An R package for phylogenetic comparative biology (and other things). Methods Ecol. Evol..

[111] Paradis E, Schliep K (2019). ape 5.0: An environment for modern phylogenetics and evolutionary analyses in R. Bioinformatics.

[112] Pagel M (1994). Detecting correlated evolution on phylogenies: A general method for the comparative analysis of discrete characters. Proc. R. Soc. Lond. Biol. Sci..

[113] Fritz SA, Purvis A (2010). Selectivity in mammalian extinction risk and threat types: A new measure of phylogenetic signal strength in binary traits. Conserv. Biol..

[114] R Core Team. R: A Language and Environment for Statistical Computing. R Foundation for Statistical Computing, Vienna, Austria (2021). https://www.R-project.org. (Accessed February 23, 2019)

