# Supplementary material for: The ultimate database to (re)set the evolutionary history of primate genital bones
Source: Sci Rep. 2021 May 27;11:11245. doi: 10.1038/s41598-021-90787-2 (PMC8160331; doi:10.1038/s41598-021-90787-2)
Supplement: Supplementary file 1 — Supplementary Legends. [file 41598_2021_90787_MOESM1_ESM.docx]

**Supplementary Information**

**Supplementary Table S1**

***Baculum* and *baubellum* literature occurrence dataset**

Dataset of primate genital bone occurrence (N=306 species) based on literature data only. Specimens are listed in alphabetical order. For each species either presence (1) or absence (0) at both species (‘P/A sp’) and genus (‘P/A gen’) levels are shown; 1/0 when conflicting data are found; *=presence of cartilage; blank cells indicate the absence of *datum* in literature. In the ‘References’ column, it is reported either the original citation for the raw occurrence data (*baculum/baubellum*) (N=45 references) or the 'inferred' status, which indicates species whose occurrence data derived from other species from which they stemmed either as previous sub-species or by a species splitting after publication time. When more than one reference is present for the same *datum*, the “/” separates references of conflictual occurrence data, while the “;” separates references related to species and genus occurrence data, respectively. The ‘Notes’ column refers to the species name originally used in the reference paper (see text for taxonomical issues).

**Supplementary Table S2**

**Fresh and museum scanned samples of primate genitals**

Primate specimen list of both fresh and museum samples of either whole bodies or external genitals (N=148 specimens representing 3 genera, 68 species and 2 subspecies). For each sample ample information was supplied: provider (AMNH - American Museum of Natural History, USA; NMNH - National Museum of Natural History, USA; NHMLS - Natural History Museum of “La Specola”, IT; MZUTT – Primatological Collection of the Torino University, IT; Istituto Zooprofilattico Sperimentale – IZS – of Piedmont, Liguria and Aosta Valley, IT – IZSTO, Trentino-South Tyrol, Veneto and Friuli-Venezia Giulia, IT – IZSVE, Lombardy and Emilia-Romagna, IT - IZSLER, Lazio and Tuscany, IT - IZSLT); identification number (ID); updated scientific name; sex; age class (with exact age, if available); sample type (either whole bodies or external genitals); occurrence data from the literature (Lit. - ?=cartilage maybe; 1‡=only occurrence data for the genus were available in the literature; 0*-1*=only inferred occurrence data were available in the literature; DD=data deficient, that is no occurrence data were available in the literature); present study data (occurrence data obtained by applying the methodological protocol showed in Table 1); taxonomical notes (old names of species if different from the updated ones).

**Supplementary Figure S3**

**Ancestral character state reconstruction of *baculum* including outgroups**

Result from 1000 stochastic character maps (where the character analyzed is the *baculum* occurrence) displayed in aggregate and outgroups included. The colour of edges in the tree gives the posterior probability (computed as the relative frequency across stochastic maps) of each *baculum* state through the history of the clade. Green indicates a high posterior probability of *baculum* presence, and numbers in green (or red) boxes indicate the proportion of iterations that mapped either a baculum presence or absence to those branches. The length of the legend also gives a scale for the branch lengths of the tree, in this case in Millions of Years Ago. Phylogeny by Springer [71], dropped. Figure generated in R (v. 4.0.3, https://www.R-project.org/) [114].

**Supplementary Table S4**

**Comparison of *baculum* and *baubellum* stochastic character mapping on different phylogenies**

Results of ancestral character state analyses performed for both *baculum* and *baubellum* and mapped on two different primate phylogenies by Springer [71] and Timetree [72]. Values of mean state change (from absence – AB – to presence – PR, and from PR to AB) and values of mean total time spent in each state (AB and PR) are provided.

**Supplementary Figure S5**

**Ancestral character state reconstruction of *baculum* on Timetree phylogeny**

Result from 1000 stochastic character maps (where the character analyzed is the *baculum* occurrence) displayed in aggregate. The colour of edges in the tree gives the posterior probability (computed as the relative frequency across stochastic maps) of each *baculum* state through the history of the clade. Green indicates a high posterior probability of *baculum* presence; red indicates a high posterior probability of *baculum* absence. The length of the legend also gives a scale for the branch lengths of the tree, in this case in Millions of Years Ago. Phylogeny by Timetree [72], dropped. Figure generated in R (v. 4.0.3, https://www.R-project.org/) [114].

**Supplementary Figure S6**

**Ancestral character state reconstruction of *baubellum* including outgroups**

Results from 1000 stochastic character maps (where the character analyzed is the *baubellum* occurrence) displayed in aggregate and outgroups included. The colour of edges in the tree gives the posterior probability (computed as the relative frequency across stochastic maps) of each *baubellum* state through the history of the clade. Green indicates a high posterior probability of *baubellum* presence, and numbers in green (or light green) boxes indicate the proportion of iterations that mapped a *baubellum* presence to those branches. The length of the legend also gives a scale for the branch lengths of the tree, in this case in Millions of Years Ago. Phylogeny by Springer [71], dropped. Figure generated in R (v. 4.0.3, https://www.R-project.org/) [114].

**Supplementary Figure S7**

**Ancestral character state reconstruction of *baubellum* on Timetree phylogeny**

Result from 1000 stochastic character maps (where the character analyzed is the *baubellum* occurrence) displayed in aggregate. The colour of edges in the tree gives the posterior probability (computed as the relative frequency across stochastic maps) of each *baculum* state through the history of the clade. Green indicates a high posterior probability of *baculum* presence; red indicates a high posterior probability of *baculum* absence. The length of the legend also gives a scale for the branch lengths of the tree, in this case in Millions of Years Ago. Phylogeny by Timetree [72], dropped. Figure generated in R (v. 4.0.3, https://www.R-project.org/) [114].

**Supplementary Figure S8**

**Comparison between this study and Brindle and Opie [27] *baculum* occurrence datasets**

*Baculum* occurrence data coverage of primate phylogeny obtained by comparing our database (left) and **Brindle and Opie [27]** database **(**right). Green lines indicate the presence of ossa genitalia. Red lines indicate the absence of ossa genitalia. Grey lines stand for ‘omitted data’ (*i.e.*, all those species for which neither absence nor presence of genital bones was ever stated). Phylogeny by Springer [71], dropped. Figure generated in R (v. 4.0.3, https://www.R-project.org/) [114].
