# Supplementary figures and images for: The ultimate database to (re)set the evolutionary history of primate genital bones
Source: Sci Rep. 2021 May 27;11:11245. doi: 10.1038/s41598-021-90787-2 (PMC8160331; doi:10.1038/s41598-021-90787-2)

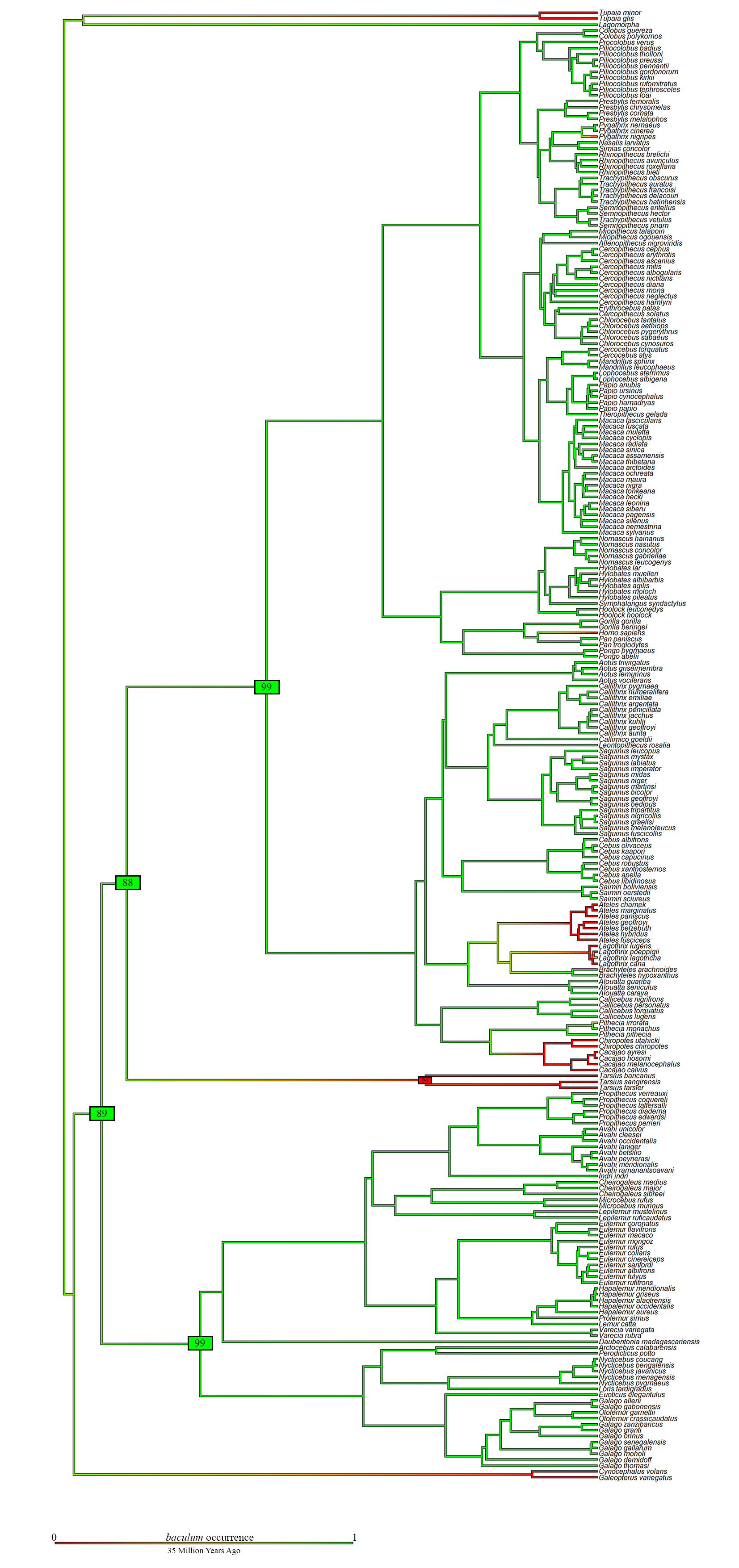

Supplement: Supplementary file 4 — Supplementary Figure S3. [file 41598_2021_90787_MOESM4_ESM.tif]

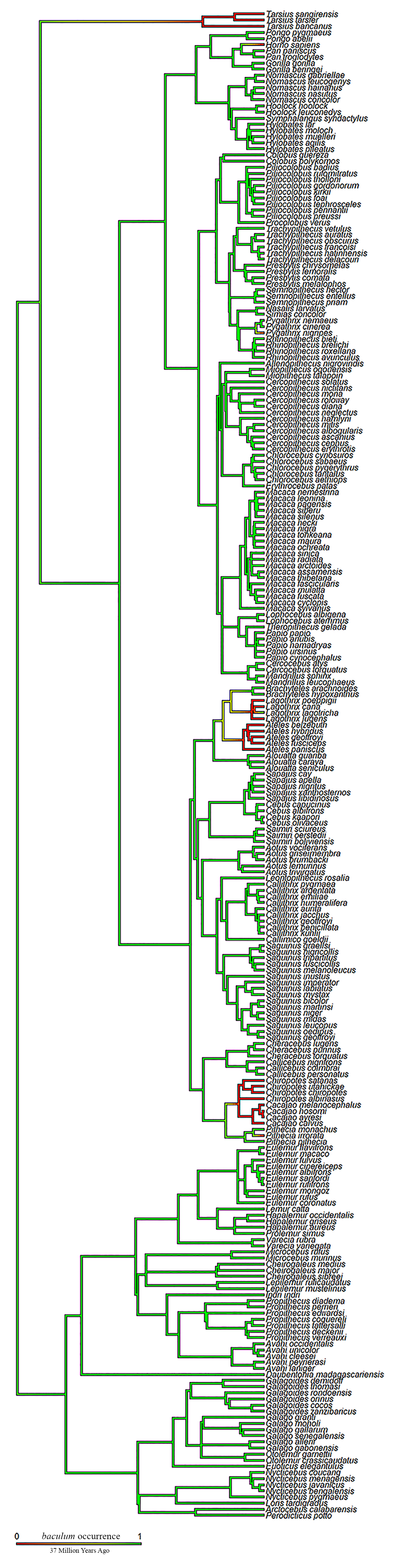

Supplement: Supplementary file 6 — Supplementary Figure S5. [file 41598_2021_90787_MOESM6_ESM.tif]

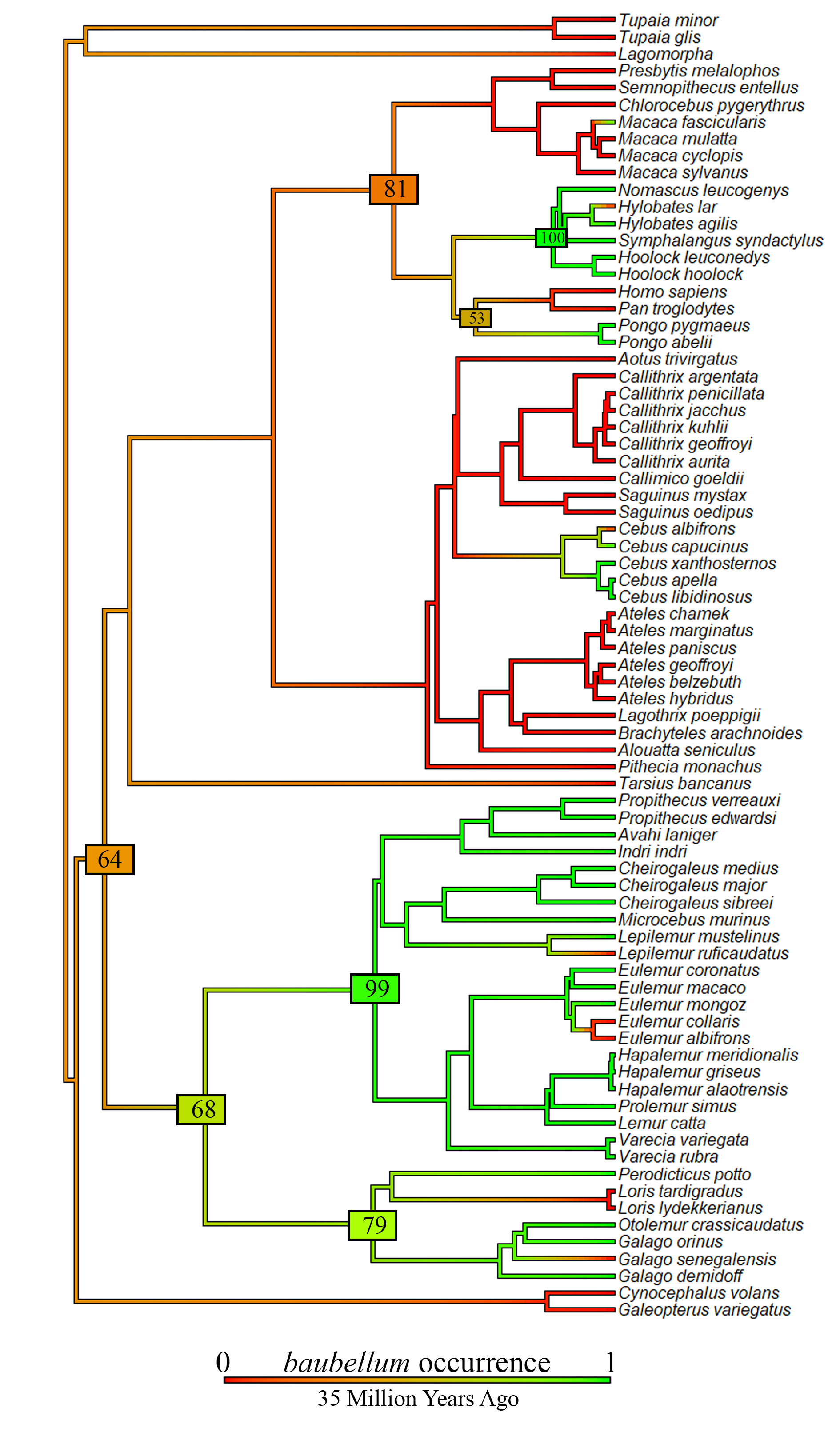

Supplement: Supplementary file 7 — Supplementary Figure S6. [file 41598_2021_90787_MOESM7_ESM.tif]

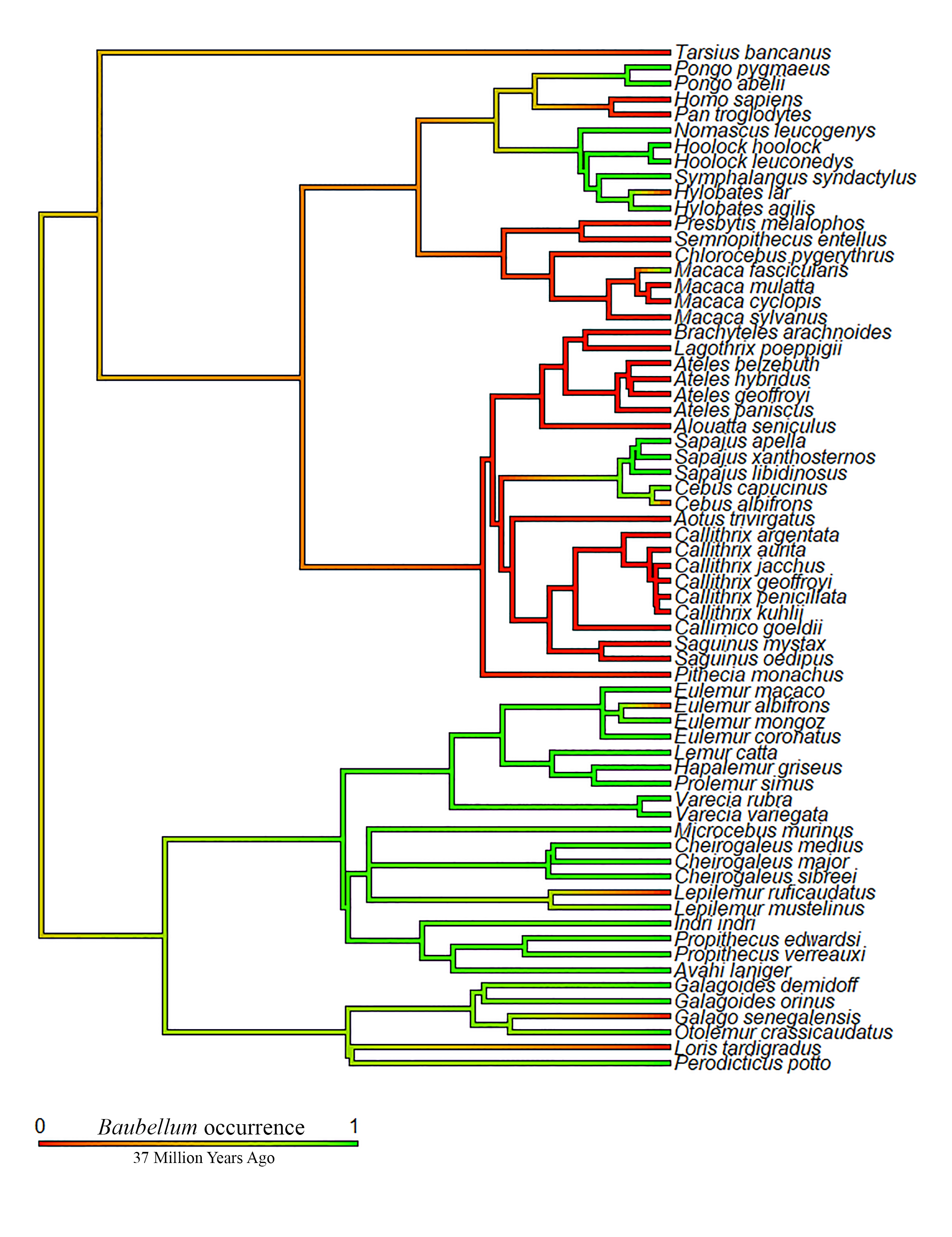

Supplement: Supplementary file 8 — Supplementary Figure S7. [file 41598_2021_90787_MOESM8_ESM.tif]

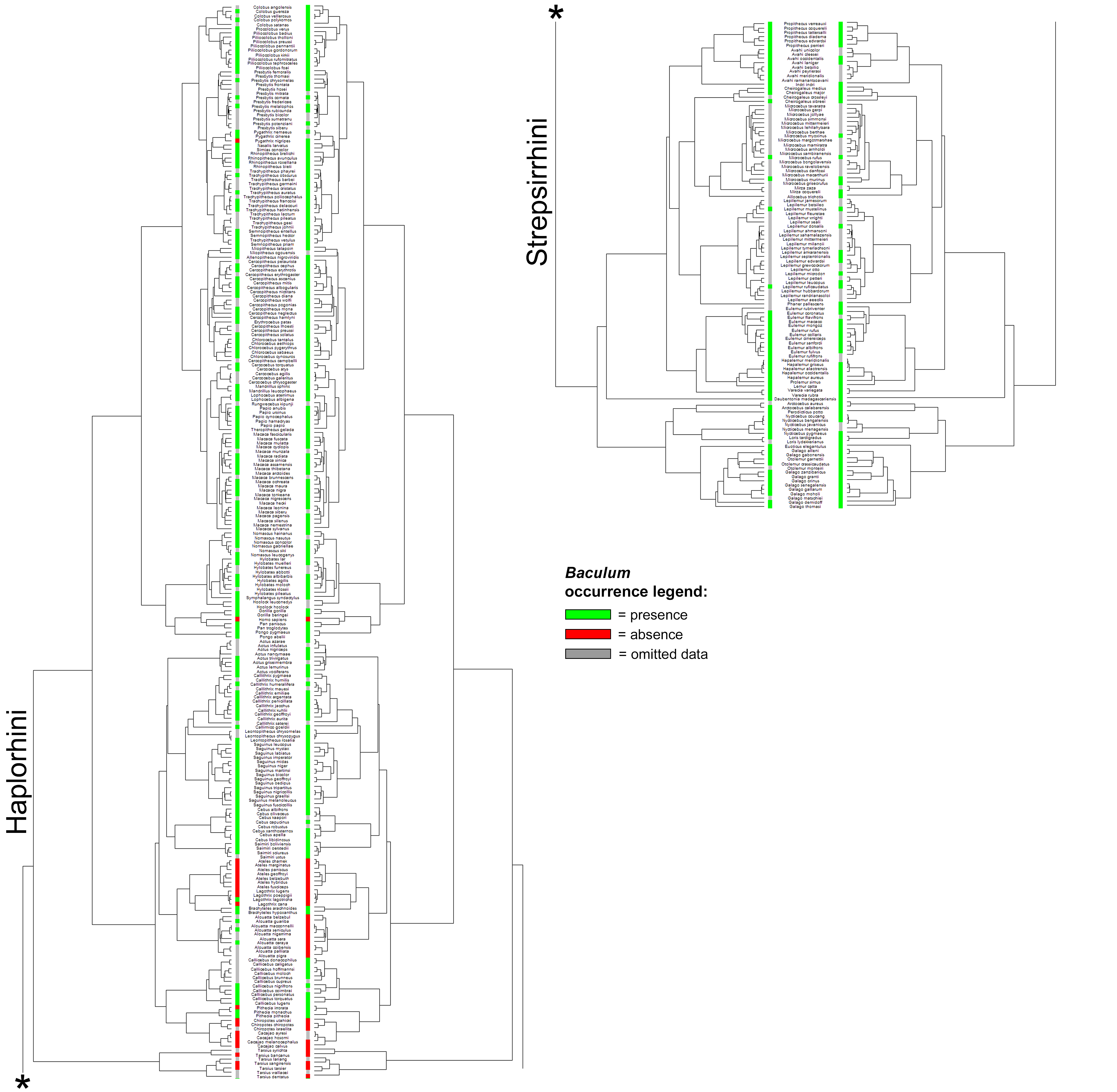

Supplement: Supplementary file 9 — Supplementary Figure S8. [file 41598_2021_90787_MOESM9_ESM.tif]
